# Metabolic Factors Limiting Performance in Marathon Runners

**DOI:** 10.1371/journal.pcbi.1000960

**Published:** 2010-10-21

**Authors:** Benjamin I. Rapoport

**Affiliations:** 1 M.D.– Ph.D. Program, Harvard Medical School, Boston, Massachusetts, United States of America; 2 Department of Electrical Engineering and Computer Science and Division of Health Sciences and Technology, Massachusetts Institute of Technology, Cambridge, Massachusetts, United States of America; University of California San Diego, United States of America

## Abstract

Each year in the past three decades has seen hundreds of thousands of runners register to run a major marathon. Of those who attempt to race over the marathon distance of 26 miles and 385 yards (42.195 kilometers), more than two-fifths experience severe and performance-limiting depletion of physiologic carbohydrate reserves (a phenomenon known as ‘hitting the wall’), and thousands drop out before reaching the finish lines (approximately 1–2% of those who start). Analyses of endurance physiology have often either used coarse approximations to suggest that human glycogen reserves are insufficient to fuel a marathon (making ‘hitting the wall’ seem inevitable), or implied that maximal glycogen loading is required in order to complete a marathon without ‘hitting the wall.’ The present computational study demonstrates that the energetic constraints on endurance runners are more subtle, and depend on several physiologic variables including the muscle mass distribution, liver and muscle glycogen densities, and running speed (exercise intensity as a fraction of aerobic capacity) of individual runners, in personalized but nevertheless quantifiable and predictable ways. The analytic approach presented here is used to estimate the distance at which runners will exhaust their glycogen stores as a function of running intensity. In so doing it also provides a basis for guidelines ensuring the safety and optimizing the performance of endurance runners, both by setting personally appropriate paces and by prescribing midrace fueling requirements for avoiding ‘the wall.’ The present analysis also sheds physiologically principled light on important standards in marathon running that until now have remained empirically defined: The qualifying times for the Boston Marathon.

## Author Summary

Marathon running, historically perceived as testing the physiologic limits of human endurance, has become increasingly popular even among recreational runners. Of those runners who test their endurance by racing the marathon distance, however, more than two in five report ‘hitting the wall,’ the rapid onset of severe fatigue and inability to maintain a high-intensity pace, resulting from the near-complete depletion of carbohydrate stores in the leg muscles and liver. An apparent paradox of long-distance running is that even the leanest athletes store enough fat to power back-to-back marathons, yet small carbohydrate reservoirs can nevertheless catastrophically limit performance in endurance exercise. In this study I develop and validate a mathematical model that facilitates computation of personalized estimates of the distances at which runners will exhaust their carbohydrate stores while running at selected paces. In addition, I provide a systematic approach to estimating personalized maximum speeds at which runners can safely complete a marathon, based on accessible physiologic parameters such as heart rate and running speed. This analysis provides a quantitative basis for improving the safety and optimizing the performance of endurance runners, evaluating midrace fueling requirements, and estimating limits of performance in human endurance running, for elite and recreational runners alike.

## Introduction

### Energy Management in Endurance Runners as a Public Health Concern

Recent years have witnessed dramatic increases in the number of amateurs participating in major endurance running events, particularly world-class marathons such as those in Boston, New York, Chicago, London, and Berlin, for which enrollment has increased by more than an order of magnitude in four decades, from hundreds of runners in the 1970s to the tens of thousands who will compete in each of the largest marathons in the 2010 season [1]. Myths and misconceptions about human physiology and how it can and should be optimized through training, nutrition, pharmacology, and performance strategy, abound in both recreational and competitive athletics, and endurance running is no exception. Endurance running severely taxes carbohydrate stores which, unlike fat reserves, can be performance-limiting because they are comparably small. Among endurance athletes, including distance runners, cyclists, and others, exhausting physiologic carbohydrate reserves is referred to as ‘hitting the wall’ or ‘bonking,’ and athletes engage in a variety of practices, collectively known as ‘carbohydrate loading,’ designed to avoid such catastrophic failure. A recent set of studies suggests that more than 40% of runners ‘hit the wall’ during a typical marathon (and that the primary risk factors for ‘hitting the wall’ are male gender, running a maximum distance of 20 miles or less during training, and expecting to ‘hit the wall’) [2], [3]. Correspondingly, energy management has traditionally been perhaps the greatest area of physiologic uncertainty in marathon running: How much carbohydrate does a given runner require to complete the race, and how can a particular runner avoid exhausting his or her carbohydrate reserves, knowing that such depletion will result in a drastic, abrupt, and painful decrease in performance?

Several investigators have analyzed the physiologic [4], [5] and energetic [6], [7] requirements of endurance running, with special attention to the marathon. A number of authors have also developed mathematical models of endurance running performance and its theoretical limitations [8]–[13], and some have applied quantitative modeling techniques to the training [14] and performance [15] of individual elite distance runners. Related experimental studies have focused on identifying performance-limiting factors in elite marathon runners [16], [17]. The principal physiologic factors contributing to endurance running performance are aerobic capacity (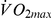) and the energetic cost of running; additional factors, such as heart morphology and lactate kinetics during exertion (in men), and adiposity and blood iron levels (in women), appear to constrain performance at the highest levels currently reached by elite marathon runners [17].

The ability of an individual runner to perform at his or her physiologic capacity, however, presupposes the availability of the metabolic fuel substrates required to sustain high levels of performance. Whereas previous work has focused on the theoretical limits of endurance running performance, or concentrated on the abilities of small samples of trained athletes, the present study provides a principled approach to determining the fuel requirements and associated performance limitations of any endurance runner over a range of distances.

### Static Limitations on Energy Expenditure in Endurance Running: Physiologic Fuel Reserves and Carbohydrate Loading

The power expended by a contracting muscle is proportional to the product of the contraction speed and the force of contraction. Metabolic power increases with contraction speed because the power developed by a contracting muscle fiber is due to myosin cross-bridge cycling within the fiber, each cycle requiring the hydrolysis of ATP. Faster contractions or more contractions per unit time require more cross-bridge cycles per unit time, corresponding to greater rates of ATP use and therefore more metabolic power. Margaria and colleagues [18] as well as other groups [19] have confirmed that the power expended by a runner increases linearly with running speed over the entire aerobic range, and therefore that the total energetic cost of running depends only on the distance run and not on running speed. (The rationale for this conclusion is that expended energy is the time integral of power, and running a given distance faster requires more power but proportionately less time, leaving the energy integral unchanged.) In particular, the energetic cost, 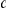, of running is approximately 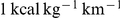, so the total energy required to complete an endurance event is 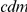, where 
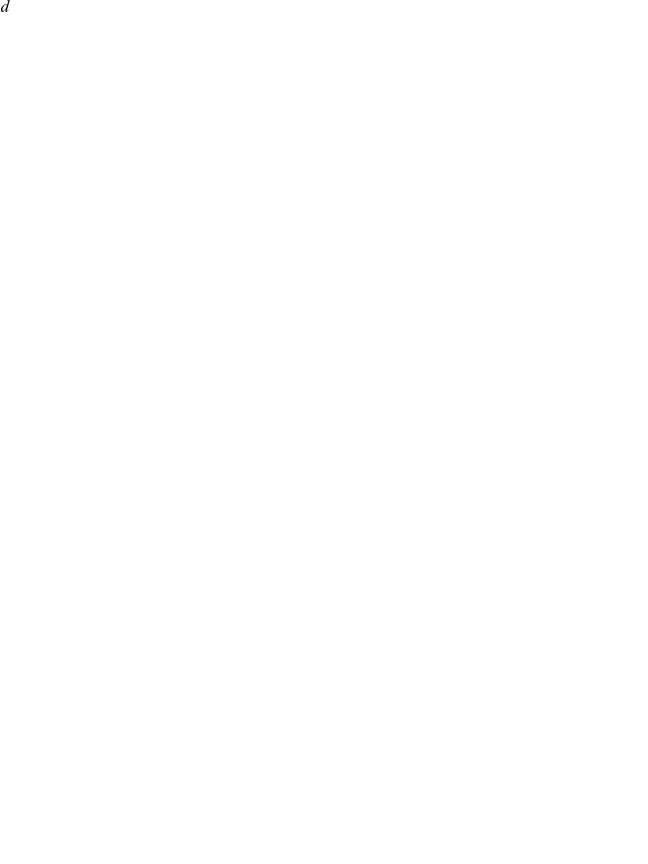
 and 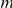 denote the distance run and the mass of the runner, respectively. For a 70-kg marathon runner, the total energy required to run a marathon, 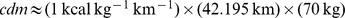, is approximately 2950 kcal. (Here 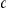 is used to approximate the total metabolic energy consumption during running, as opposed to the net excess energy consumption above the resting metabolic rate; as discussed in the Methods section, the difference is small and the present choice simplifies the modeling equations. Similarly, 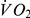 and 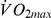 refer here to the total aerobic power and total aerobic capacity, respectively.)

Muscular contractions can be fueled by a variety of metabolic substrates, most important of which, in the context of long-distance running, are carbohydrate, derived from liver and muscle glycogen as well as from plasma glucose, and fat, including intramuscular triacylglycerols and plasma free fatty acids liberated from adipose tissue. An apparent paradox of endurance sports is that even the leanest athletes store enough metabolic potential energy to power multiple, back-to-back marathons, if only the working muscles could derive their power exclusively from fat. In terms of potential energy, a runner with nonessential body fat percentage 
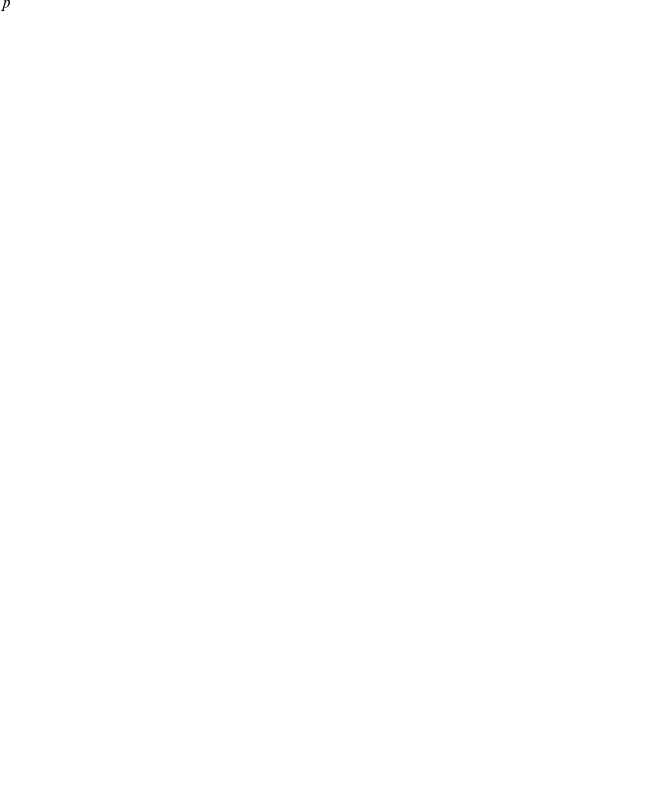
 stores enough fat to fuel a race of distance 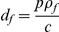 (independent of the mass of the runner), where 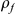 denotes the energy density of fat, which is approximately 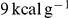. Even at the extreme lower limit of 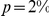 nonessential body fat, 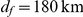, or more than four marathons. In contrast, physiologic carbohydrate stores are severely limited.

The body stores a small amount of carbohydrate in the form of plasma glucose. But as typical plasma glucose concentrations are in the 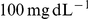 range, typical blood volume is approximately 5 L, and the energy density of carbohydrate is approximately 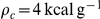, the blood plasma typically stores less than 20 kcal of glucose and is therefore essentially negligible as a carbohydrate reservoir in endurance exercise.

The liver typically stores glycogen at a density, 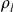, of approximately 270 mmol glycosyl residues per kilogram (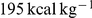), and is capable of storage at a maximum density of approximately 500 mmol glycosyl residues per kilogram (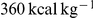). Therefore, a 1.8-kg liver typically stores approximately 88 g of carbohydrate and can store at most approximately 160 g, corresponding to approximately 350 and 650 kcal, respectively [20], [21].

Muscles store glycogen as well, but only for local use. Whereas liver glycogen can be made globally available to metabolically active cells (including working myocytes) throughout the body via glycogenolysis and release of the resulting glycogen-derived glucose into the bloodstream, muscle glycogen can be used only by the cell in which it has been synthesized and stored. The reason for this difference between liver and muscle glycogen reserves is that myocytes, in contrast to hepatocytes, lack the enzyme glucose-6-phosphatase that catalyzes the final reaction of glycogenolysis and permits membrane glucose transporters to liberate intracellular glucose. As a result, glycogen stores of the specific muscles to be used in an endurance event must be loaded prior to exercise. Optimization strategies for ‘carbohydrate loading’ abound, and not all of those used by athletes are based on sound physiologic reasoning. Several schemes have proven effective and have been reviewed by McArdle and colleagues [22]. All of these techniques are variations on a three-phase theme: Prolonged or high-intensity exercise of the muscles to be loaded, typically followed first by a period of dietary carbohydrate restriction, and then ultimately by a period of high carbohydrate intake. Such schedules are designed to induce a ‘glycogen supercompensation’ effect, whereby glycogen depletion and carbohydrate restriction stimulate increased expression of glycogen synthase in the depleted muscle fibers, enhancing their ability to synthesize glycogen during the final, high-carbohydrate-diet phase, permitting muscle fibers to store glycogen in supranormal concentrations. Exercise-induced suppression of insulin and muscle-contraction-induced activity of muscle glucose transporters also facilitate glycogen loading specifically in the target muscles, in preference to fuel storage in other physiologic energy stores such as adipose tissue and nonworking muscles. Biopsy studies of leg muscles loaded in this way indicate that while the muscles of trained athletes typically store glycogen at a density, 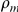, of approximately 110 mmol glycosyl residues per kilogram (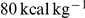), glycogen loading protocols can increase that density to a maximum of approximately 200 mmol glycosyl residues per kilogram (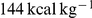) [23]. While the maximum size of the glycogen reservoir available to an endurance runner depends on the size of the relevant muscles, it is possible to estimate the amount of accessible glycogen: A lean, male runner, for example, may be 45% skeletal muscle by mass, with half of that mass in his leg muscles; at 70 kg such an athlete would typically store 310 g of carbohydrate as muscle glycogen, and could store at most approximately 570 g, corresponding to approximately 1250 and 2270 kcal of leg muscle glycogen, respectively.

Considering the total carbohydrate-based energy reserve from muscle glycogen, liver glycogen, and plasma glucose, it becomes clear that normal carbohydrate stores alone would be insufficient to fuel a marathon. Furthermore, only in the glycogen-loaded state, in which glycogen reserves approach their physiologic capacity, can the levels of stored carbohydrate approach those necessary to power a marathon by carbohydrate alone. However, the foregoing, conventional approach to accounting for physiologic potential energy reservoirs reveals only the static part of the story of energy management in marathon running. In addition to these static considerations there are also important dynamic ones, modeled in the present work; however, endurance athletes and those who advise them sometimes neglect the dynamics of fuel metabolism and consequently miscalculate their fuel requirements.

### Personalized Physiologic Modeling for Safe and Optimal Performance in Marathon Running

The energy concerns of endurance runners center principally around two questions: How much carbohydrate does a particular runner need to race over a given distance, and How can each runner be sure to avoid exhausting his or her carbohydrate reserves before completing the race? By synthesizing and quantitatively analyzing human physiologic data collected in studies spanning the last several decades, the present work derives a modeling framework for providing personalized answers to these questions. The model takes into account aerobic capacity, which can be measured by conventional protocols or estimated on the basis of heart rate response to running at known speeds; relative exertion, or fraction of total aerobic capacity at which the race is run (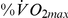), which can be determined from running speed in an individual runner with known aerobic capacity; and the size of the glycogen reservoir under typical and maximally loaded conditions, which depends on the distribution of the leg musculature in an individual runner and can be estimated as a function of body mass. The model incorporates the metabolic cost of running and the known dependence of fat versus carbohydrate metabolism on relative exertion. This work reveals the functional dependencies of the distance required to run glycogen stores to depletion, as well as the fastest pace at which a given distance, such as the marathon, can be run without exhausting glycogen stores. It also provides a quantitative approach to establishing an effective midrace fueling strategy, designed to extend the distance a given runner can cover, or to increase the maximum pace at which a runner can cover a given distance, before exhausting his or her glycogen stores. In addition, the present work shows how individual physiologic variation as well as the population distribution of aerobic capacities limit marathon finishing times, providing a principled basis for marathon qualifying standards such as those used in the Boston Marathon, which until now have only been empirically determined.

## Results

### Aerobic Exercise Intensity Determines Relative Usage of Fat and Carbohydrate as Fuel Substrates During Running

Working muscles consume a mixture of metabolic substrates, and the relative contributions of fat and carbohydrate to this mixture dynamically depend on exercise intensity and the size of available glycogen reservoirs: Carbohydrates account for a greater proportion at higher intensities, while fat accounts for a greater proportion as available glycogen is depleted [24]. These trends reflect the significantly greater efficiency of carbohydrate relative to fat as a fuel for aerobic exercise, as discussed in greater detail in the Methods section: Carbohydrate oxidation typically generates approximately 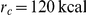 per mole of respired oxygen, whereas fatty acid oxidation typically generates only approximately 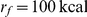 per mole of oxygen. As a consequence, total carbohydrate consumption over the course of a marathon, and therefore the crucial question of whether the body can store enough carbohydrate fuel to complete the race, depends not only on the distance to be run but also on the intensity at which the race is run. Moreover, the rate at which ATP can be generated through physiologic processes depends on both the fuel substrate and the reaction end product (carbon dioxide in aerobic processes, regardless of the substrate; lactate or creatine in anaerobic glycolysis or hydrolysis of phosphocreatine, respectively). More precisely, there is a hierarchy of metabolic processes, defined by the rate at which ATP can be produced to power muscle contractions: The anaerobic processes, hydrolysis of phosphocreatine and conversion of glycogen to lactate, produce at most 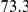 and 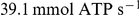, respectively; by contrast, the aerobic processes, which involve the complete oxidation of muscle glycogen, liver glycogen, or adipose-tissue-derived fatty acids, produce at most 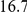, 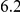 or 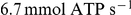, respectively [25]. The maximal rate of ATP extraction tends to decrease as the size of the fuel reservoir increases.

Such considerations of substrate and efficiency underscore the importance of adequate carbohydrate reserves for endurance runners. Low glycogen and plasma glucose levels during exercise lead to an elevated ratio of glucagon to insulin, promoting lipolysis and the release of fatty acids from adipose tissue. In active muscle, fatty acids undergo 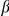-oxidation to acetyl CoA and eventually carbon dioxide. The resulting elevated levels of acetyl CoA partially suppress carbohydrate metabolism, reducing the flux of pyruvate into the citric acid cycle by inhibiting the conversion of pyruvate to acetyl CoA [25]. This biochemical feedback network forestalls complete glycogen depletion, but simultaneously decreases the energy efficiency of oxygen utilization.

**Figure 1:**
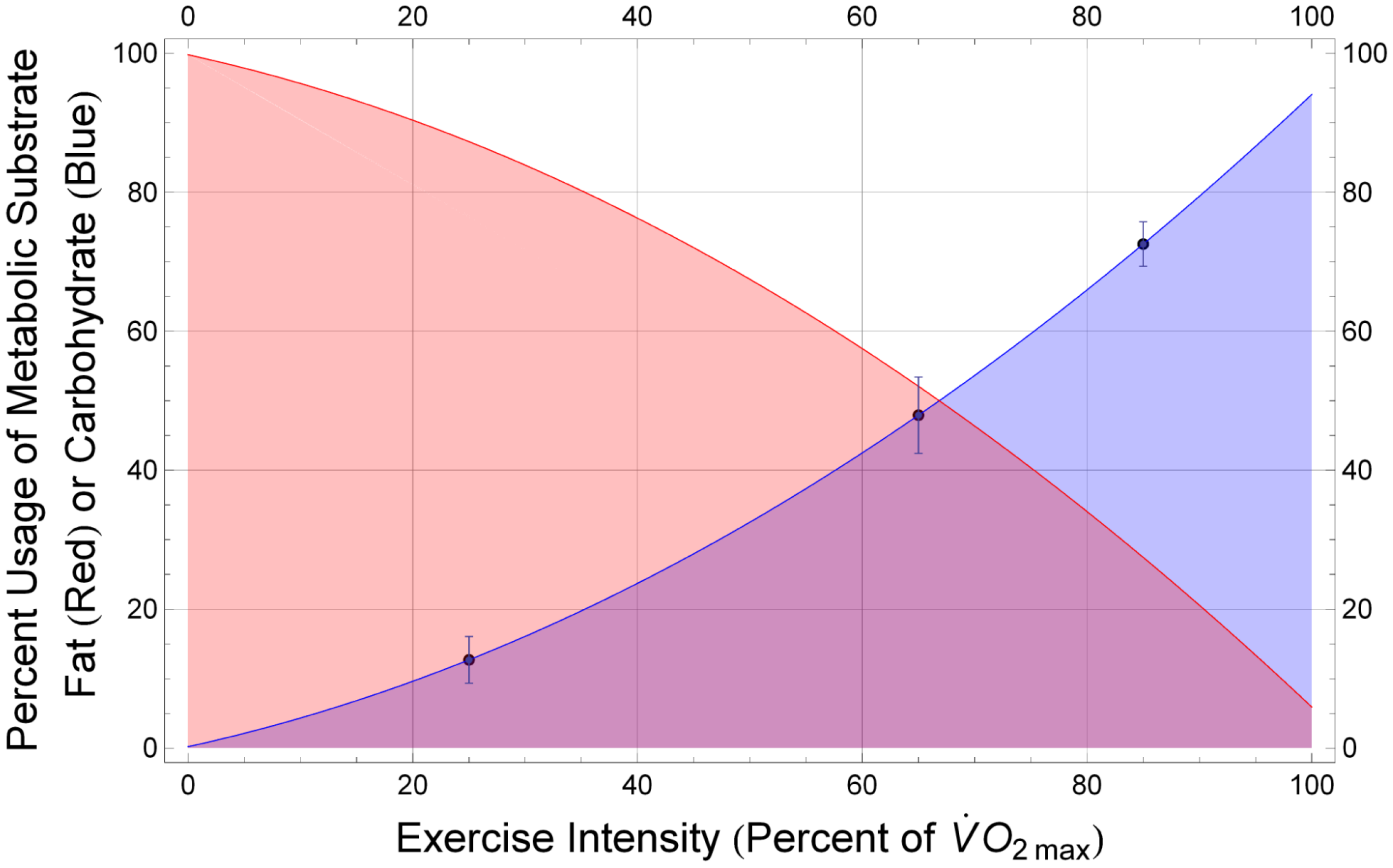
Relative use of fat and carbohydrate as metabolic fuels depends on exercise intensity. Fractional usage of carbohydrate (plasma glucose plus muscle glycogen, blue filled curve, 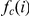) and fat (plasma free fatty acids plus muscle triglycerides, red filled curve, 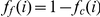) are shown as functions of relative exercise intensity, 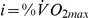. (Based on the work of Romijn and colleagues: Points plotted correspond to data points from the 1993 study [24], and corresponding error bars are computed as described in the Methods section.)

The work of Romijn and colleagues [24] has made it possible to estimate the composition of the metabolic mixture consumed during exercise as a function of exercise intensity, as discussed in the Methods section: Figure 1 shows fractional usage of carbohydrate (plasma glucose plus muscle glycogen, 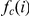) and fat (plasma free fatty acids plus muscle triglycerides, 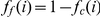) as functions of relative exercise intensity, 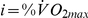. These functions and the stoichiometry of muscle oxygen metabolism, reflected in the parameters 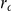 and 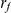, permit the expression of 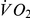 in terms of power output as in Equation 1, derived in the Methods section.

### Individual Physiologic Parameters and Aerobic Exercise Intensity Determine Maximum Safe Running Speeds Over Endurance Distances

The computational approach presented here can be used to estimate the total carbohydrate consumption of a marathon runner over the course of a race, as discussed in detail in the Methods section, where Equation 3 is derived to express the maximum aerobic running speed of an arbitrary runner. Whether the total amount of carbohydrate available to that runner will suffice to fuel his run at the chosen pace, however, depends on the sizes of his liver and working muscles relative to his total body mass (the total mass whose movement their glycogen stores must power), and on the density at which they have been loaded with glycogen prior to the run, as described in detail in the Methods section.

**Figure 2:**
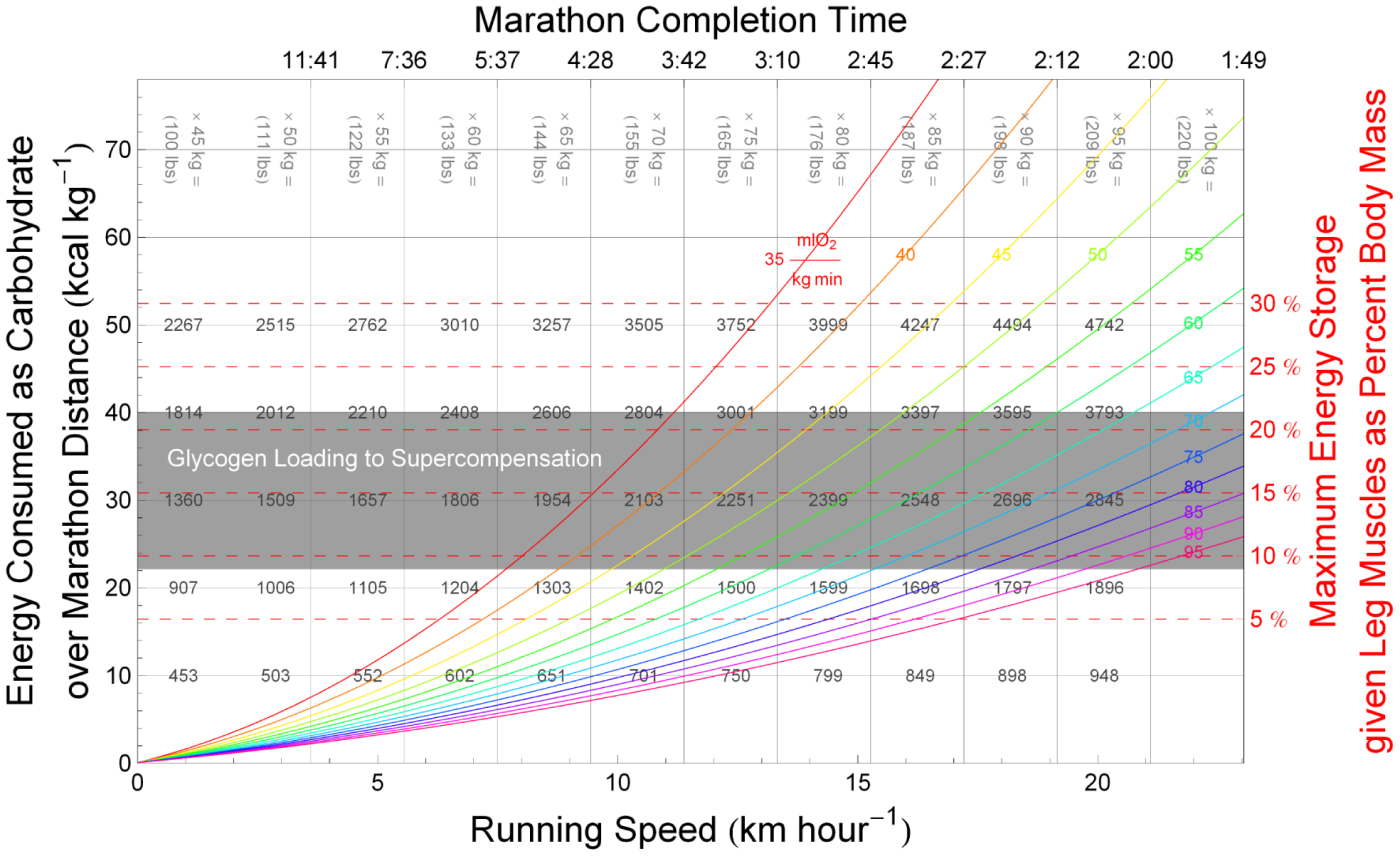
When does glycogen storage capacity constrain maximal marathon speed? Computed approximations of total energy consumed as carbohydrate over the course of a marathon, as a function of running speed, in runners with various aerobic capacities. Each colored line corresponds to a particular value of aerobic capacity, 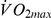, in terms of milliliters of oxygen per kilogram body mass per minute, as labeled (Dark Orange, 35; Orange, 40; Yellow, 45; Light Yellow, 50; Light Green, 55; Green, 60; Aqua, 65; Light Blue, 70; Blue,75; Purple, 80; Violet, 85; Magenta, 90; Red, 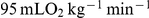). The vertical scale is expressed in terms of kilocalories of energy consumed per kilogram of body mass over 42.195 km (26 miles and 385 yards), the length of a marathon; the corresponding total energy consumption for runners of various masses is shown along the horizontal trend lines, beneath the values of body mass labeled along the top edge of the plot. Running speed is expressed in kilometers per hour along the lower horizontal axis, and as total time to complete a marathon at the corresponding speed along the upper horizontal axis. The dashed, horizontal red lines show the estimated maximum energy storage capacity in runners with maximally glycogen-loaded livers and glycogen-loaded muscles in a state of maximal glycogen supercompensation (144 kcal glycogen per kilogram leg muscle), for whom the leg muscles powering running constitute the indicated percentages of total body mass (right-sided vertical axis). The shaded region indicates the range of supranormal energy storage capacities available to a typical male runner, whose leg muscles constitute approximately 21.4% of his total body mass; the boundaries of the shaded region correspond to typical and maximal values of muscle glycogen density for trained endurance athletes (80 and 144 kcal glycogen per kilogram leg muscle, respectively). See the text for a detailed explanation.

Figure 2 illustrates the dependence of total carbohydrate usage on running speed for marathon runners (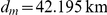 (26 miles and 385 yards)) with a range of body masses and aerobic capacities, along with the constraints imposed by total body glycogen storage capacity derived in the Methods section. For a runner of given aerobic capacity, the fastest possible marathon pace corresponds to the horizontal position of the point at which the appropriate colored curve in Figure 2 intersects the horizontal line indicating his or her relative leg muscle mass (several exemplary dashed red lines are drawn from the right-hand vertical axis): Runners with large aerobic capacities and relatively large leg muscles can store enough liver and muscle glycogen to fuel marathon runs at elite-athlete paces (paces approaching those required to challenge the current world records of 2:03:59 for men and 2:15:25 for women) without exhausting physiologic carbohydrate stores; runners with smaller aerobic capacities or relatively small leg muscles must run at slower paces or refuel during the race in order to avoid ‘hitting the wall.’

For example, Figure 2 indicates that a runner with a 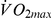 of 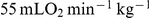 (corresponding to the light green curve) requires approximately 20 kilocalories per kilogram of body mass to complete a marathon in 3:42:00 (the horizontal grid line corresponding to 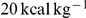 intersects the vertical grid line for a marathon finishing time of 3:42:00 on the light green curve). The numbers along the horizontal grid line for 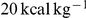 indicate total energy derived from carbohydrates over the course of the race for runners of varying body mass; if the runner in question weighs 75 kilograms, for example, his glycogen stores must total at least 1500 kilocalories to ensure that he can complete the race without ‘hitting the wall’ (assuming he consumes no carbohydrate during the race). As this rate of energy expenditure falls below the shaded zone indicating ‘Glycogen Loading to Supercompensation,’ the runner can be confident that a target pace of 3:42:00 (corresponding to approximately 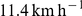 or 8:29 per mile) is physiologically sustainable given his aerobic capacity, provided that his leg muscles constitute at least 7.5% of his body mass (as indicated by the dashed red lines from the right-sided vertical axis). On the other hand, if the runner in question has a 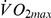 of only 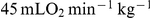 (corresponding to the yellow curve), he may not be able to sustain an 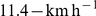 marathon pace: the intersection of the yellow curve with the vertical 3:42:00 grid line occurs within the ‘Glycogen Loading to Supercompensation’ region, indicating that the runner must implement a carbohydrate-loading strategy in preparation for the race; moreover, the position of the intersection point relative to the right-axis grid lines indicates that even maximal carbohydrate loading would be insufficient to power a 3:42:00 marathon for this runner unless his leg muscles constitute at least approximately 12.5% of his body mass.

The predictions of the model presented here can be validated through comparison with direct experimental observations on competitive runners. Karlsson and colleagues [26] studied a group of ten runners who ran the same 30-kilometer race twice, three weeks apart, under carbohydrate-loaded and -unloaded conditions. As described in the Methods section, Equation 6 can be used to predict the changes in muscle glycogen density in each runner (measured in biopsy studies by Karlsson and colleagues) as a function of his physiologic parameters (body mass and aerobic capacity) and average racing speed. The predictions of the model are consistent with the observations of Karlsson and colleagues. In particular, assuming that runners consumed no exogenous carbohydrate during the races, the mean ratio of model-predicted to experimentally observed decrease in leg muscle glycogen density is 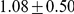 (standard error); under the opposite assumption that runners consumed the maximum allowable amount of exogenous carbohydrate during the races, the mean ratio of modeled to predicted change in glycogen density is 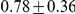. (In both cases perfect agreement would correspond to a ratio of unity.)

### Maximal Fuel Economy in Endurance Running is Achieved at Constant Levels of Exertion

Reconsidering the data shown in Figure 1 in light of the foregoing analysis reveals why running at a constant speed, as assumed in the present discussion, is the most metabolically efficient pacing strategy for completing an endurance race in a given time. (The critical parameter, in fact, is the level of exertion, 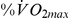, which over a uniform course is approximately proportional to speed for an individual runner; over courses complicated by features such as hills or variable terrain, however, this simple relation can break down, in which case maintaining a constant level of exertion, rather than a constant pace, is most metabolically efficient. The functional dependence of 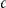 on ground incline has been described by Margaria and colleagues [18], among others.) Although total energy expended is independent of speed and depends only on the distance run, the proportional contribution of carbohydrate to the metabolic mixture used by a given runner increases supralinearly with the speed of that runner. If a runner wishes to complete a race over a distance 
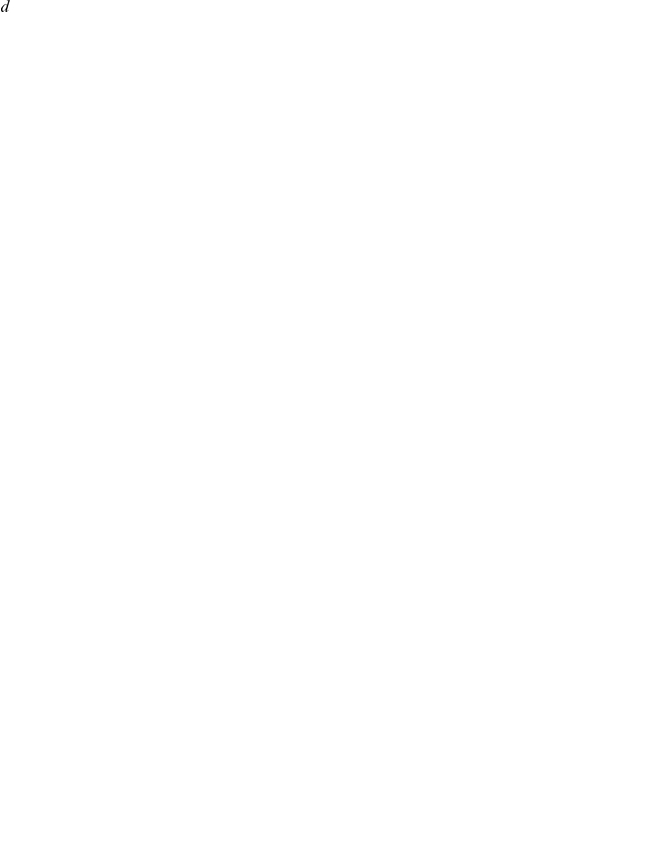
 in total time 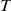, she must maintain an overall average pace of 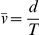. If she falls below this target pace for any interval of time, she must compensate later by running faster than 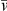 in order to maintain her desired average speed over the entire distance. During the slow interval, her carbohydrate consumption falls below what it would have been had she maintained her target pace; however, during the compensatory fast interval, her carbohydrate consumption not only exceeds what it would have been had she maintained her target pace, it is also greater in magnitude than the carbohydrate savings she achieved during the slow interval. As a result, her net carbohydrate consumption is greater than it would have been had she never deviated from her target pace.

### Demographics of Marathon Finishing Times and the Risk of ‘Hitting the Wall’

The present analysis is consistent with findings from population studies of marathon runners, and also sheds light on important standards in marathon running that until now have remained empirically defined: the principal qualifying times for the Boston Marathon.

The modal finishing time in large marathons open to all runners is between four and five hours. Figure 2 illustrates that the typical distribution of marathon finishing times, as presented by Sabhapandit and colleagues [27], is consistent with the population distribution of 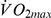, which falls between approximately 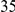 and 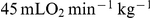 for men below the age of 50 (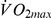 is systematically lower among women, and declines with age in men and women) [28]. Typical levels of glycogen loading permit runners with 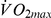 between approximately 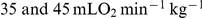 to complete a marathon safely in between four and five hours, as indicated by the intersections of the corresponding curves with the lower border of the ‘Glycogen Loading to Supercompensation’ region in Figure 2.

Buman and colleagues have shown that the likelihood of ‘hitting the wall’ during a marathon exhibits a peak around mile 21 (kilometer 33–34), followed by a sharp decline [2], [3]. Popular accounts of endurance running often either argue through approximation that physiologic human glycogen reserves are insufficient to fuel a marathon (propagating the myth that ‘hitting the wall’ is inevitable), or imply that maximal glycogen loading is a universal requirement for runners attempting to complete a marathon. In contrast, the present work demonstrates that the energetic constraints on endurance runners are more subtle, depending on several physiologic variables including the relative leg muscle mass, liver and muscle glycogen density, and running speed (exercise intensity as a fraction of aerobic capacity) of individual runners, in personalized but nevertheless quantifiable and predictable ways. Consistent with the findings of Buman and colleagues, the analytic approach presented here can be used to estimate the distance at which a runner will exhaust his glycogen stores (‘hit the wall’) as a function of these physiologic parameters.

**Figure 3:**
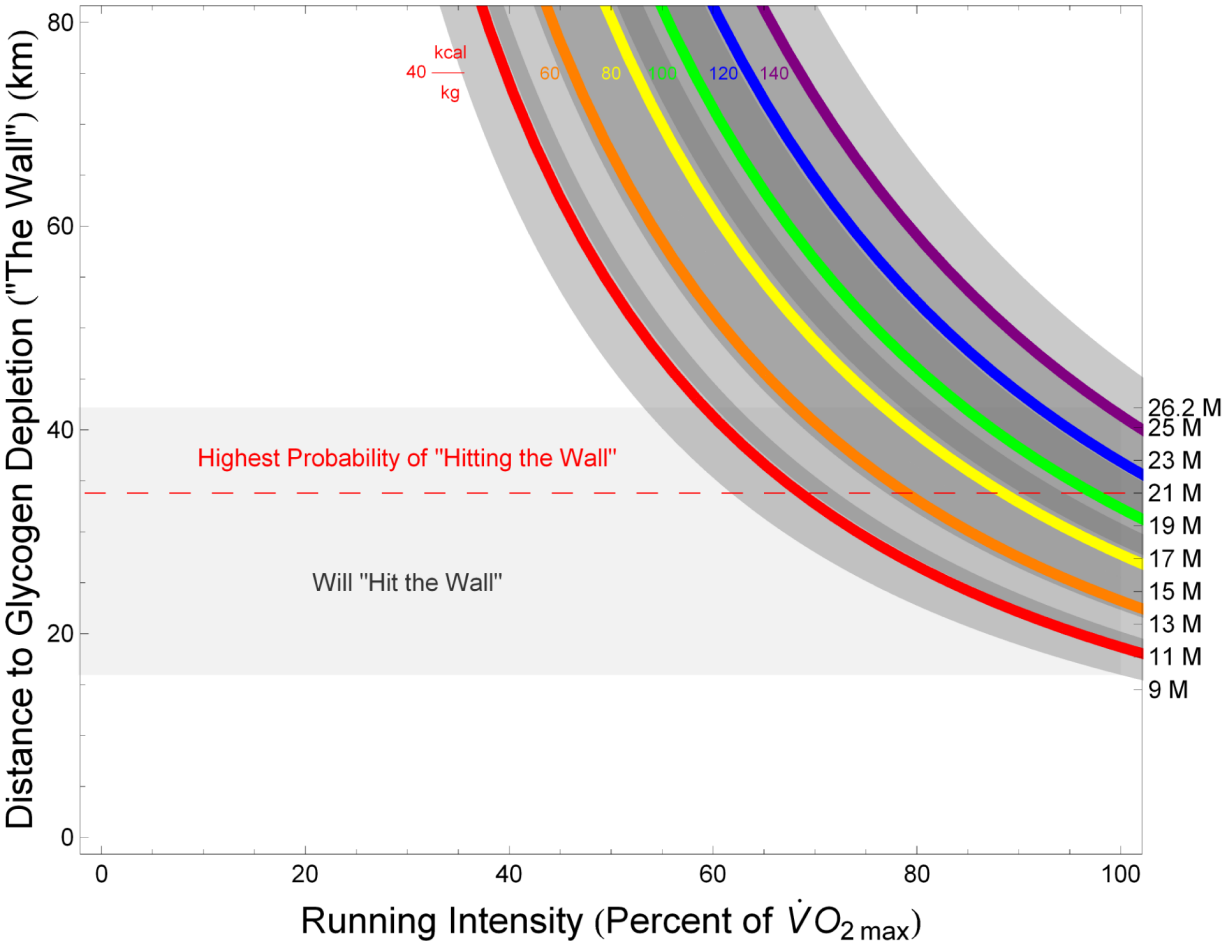
Distance to ‘The Wall’ for endurance runners. Computed distance athletes can run before completely depleting glycogen reserves (‘hitting the wall’), as a function of running intensity (expressed as a percent of 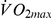), relative leg muscle mass (leg muscle mass as a fraction of total body mass), and muscle glycogen density. The distance an athlete can run before ‘hitting the wall’ decreases with increasing levels of exertion, and as the shaded rectangular region labeled ‘Will “Hit the Wall”’ indicates, if this distance is less than 42.195 kilometers (26 miles and 385 yards) then the runner will not be able to complete a marathon without experiencing complete glycogen depletion (at least not without refueling midrace). Each colored curve corresponds to a particular density of muscle glycogen, as labeled (Red, 40; Orange, 60; Yellow, 80; Green, 100; Blue, 120; Purple, 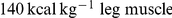). The colored curves each correspond to athletes whose leg muscles constitute 21.4% of total body mass and are loaded with glycogen at a particular density (relative liver mass has been assumed constant at 2.5% of total body mass, and liver glycogen density has been assumed maximized at 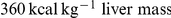); a shaded region around each colored curve fills the region corresponding to relative leg muscle masses of 15% to 25% total body mass. Darker shading indicates overlapping regions and identical failure distances for different sets of physical parameters: The area of densest shading straddles the 21-mile line for athletes running at intensities of 80% to 95% 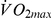, indicating that many different athletic builds and levels of glycogen loading are subject to failure at these intensities around mile 21, which has been empirically identified as the distance at which marathon runners most commonly ‘hit the wall.’ By contrast, few runners will ‘hit the wall’ before mile 11, or when running a marathon at less than 55% 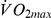.

As discussed in detail in the Methods section, where Equation 5 is derived to express the distance, 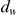, an athlete can run before ‘hitting the wall,’ 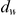 declines with increasing levels of exertion (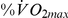), and with decreases in relative leg muscle mass or leg muscle glycogen density. However, because the latter two parameters contribute as a product, relative decreases in one can be offset by inversely proportional increases in the other, so many different sets of physical parameters can result in glycogen depletion at a particular distance. In particular, as indicated by the region of densest shading among the curves in Figure 3, athletes with a very broad range of leg muscle builds and muscle glycogen densities, when running at intensities of 80% to 95% 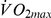, are subject to failure (defined by the condition 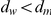) at around mile 21.

The distribution of 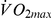 in the general male population falls between approximately 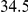 and 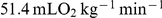 (10th and 90th percentiles, respectively), for men aged 20 to 29 years [28], and has been shown capable of increasing to a maximum of approximately 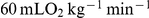 with endurance training [29]. For runners of typical builds with glycogen stores loaded according to ordinary training regimes, this aerobic capacity is marginally insufficient to run a marathon at the pace (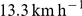, or 7:15 per mile) required to finish in 3 hours 10 minutes, the current principal male qualifying time for the Boston Marathon, entrance into which is considered a mark of distinction among amateur runners. This situation can be inferred from Figure 2, in which the green curve corresponding to a 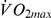 of approximately 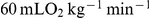 intersects the lower limit of the shaded ‘Glycogen Loading to Supercompensation’ region at a pace slightly slower than 3:10 (equivalently, the curve intersects the vertical grid line corresponding to a 3:10 pace inside the ‘Supercompensation’ regime), indicating that typical physiologic glycogen stores are marginally insufficient to power a marathon at the Boston-qualifying-time pace. The typical male runner hoping to run a qualifying time for the Boston Marathon must therefore either achieve some degree of supranormal glycogen loading (through a glycogen supercompensation protocol prior to the race) or strategically refuel during the race, as described in the Methods section.

The situation is similarly challenging for female runners. The distribution of 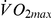 in the general female population falls between approximately 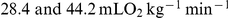 (10th and 90th percentiles, respectively), for women aged 20 to 29 years. Allowing for a training-induced relative increase in 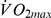 equal to that observed in male runners yields an approximate 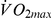 ceiling of 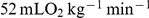 for typical female runners. Referring to Figure 2, this value of 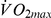 falls between the light yellow and light green curves, intersecting the lower boundary of the ‘Glycogen Loading to Supercompensation’ regime at a pace corresponding to a marathon finishing time of approximately 3:40, which is the Boston Marathon qualifying time for women in the 18-to-34–year age group.

### Midrace Fueling Strategies

Equations 5 and 7, derived in the Methods section, can be used as the basis for a personalized fueling strategy to run a given distance without ‘hitting the wall.’ In the case of a marathon runner, one first applies Equation 5 to compute 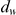, the distance to ‘the wall’ (glycogen depletion) for the runner in question given his or her physiologic parameters and intended pace. If 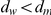, the runner cannot complete the race without ‘hitting the wall’ unless he or she adopts a fueling strategy en route. Equation 7 can then be applied, setting 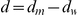, to determine the minimum amount of carbohydrate that must be consumed over the course of the race to ensure that the runner can maintain his or her target pace without exhausting his or her glycogen stores.

## Discussion

The present study demonstrates that glycogen storage capacity is only a performance-limiting factor in runners of low and moderate aerobic capacities, or with relatively small leg muscles. By contrast, elite long-distance runners typically have large aerobic capacities (above 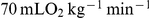), lean upper bodies, and relatively large thigh muscles. For such runners, according to the analysis described here and summarized graphically in Figures 2 and 3, glycogen storage capacity should not limit record marathon times to those established on major courses across the world, which presently hover around 2:04. Many investigators have suggested that additional sources of metabolic dynamics, such as lactate kinetics during exertion, constrain performance at or beyond the fastest paces currently maintained by elite marathon runners [10], [11], [16], [17].

Carbohydrate loading prior to marathon running influences performance because it permits a runner of a given aerobic capacity (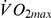) and leg muscle distribution to run at greater speeds without ‘hitting the wall,’ succumbing to the failure mode associated with the exhaustion of glycogen reserves. Effective midrace fueling through consumption of carbohydrates while running can similarly contribute to performance: Adding exogenous carbohydrates as a fuel source during a race can enable runners to maintain paces closer to their maximum aerobic speeds without exhausting their glycogen reserves. The present exposition explicitly derives a personalized prescription for the minimum amount of supplemental carbohydrate that must be consumed during a race to avoid ‘hitting the wall.’ Similarly, the model presented here can be used as a framework for estimating the amount of exogenous carbohydrate required to sustain a desired increase in speed in long-distance events such as the marathon.

The degree to which glycogen stores can be spared by introducing exogenous carbohydrate into the plasma reservoir is not entirely clear. Continuous exercise draws carbohydrate from both the plasma (replenished from liver stores and exogenous sources) and intramuscular reservoirs. The relative contribution of intramuscular glycogen to total energy expenditure appears to depend in part on relative reservoir size [30]. Experimental evidence also suggests that midrace fueling extends endurance capacity by increasing the proportion of oxidized carbohydrate derived from plasma glucose as opposed to intramuscular glycogen [31], and correspondingly reducing the rate of intramuscular glycogen depletion [32]. Oxidation of intramuscular glycogen does not appear to be completely suppressed by midrace carbohydrate consumption, however; at least at high intensities, prerace muscle glycogen storage capacity appears to remain a performance-limiting factor over long distances.

The form in which supplemental carbohydrate is consumed can influence the effectiveness of midrace fuelings. Ingested carbohydrate is useful only to the extent that it becomes available to the working muscles before the onset of glycogen depletion. Following ingestion, the source of carbohydrate must be released by the stomach into the small intestine for absorption into the bloodstream. The rate at which fluids exit the stomach has long been known to exhibit a strong dependence on osmolarity, being most rapid for isotonic fluids [33]. Consistent with this physiologic phenomenon, a number of studies have confirmed that consumption of carbohydrate-containing beverages during endurance exercise can delay the onset of fatigue [22], [34], and commercial carbohydrate-electrolyte beverages designed for the athletic market tend to be approximately isotonic. The timing and distribution of midrace fuelings evidently have little impact on their effectiveness, provided the required total amount of carbohydrate is consumed sufficiently far (typically approximately 30 minutes) in advance of the anticipated onset of fatigue [22].

The analysis presented here provides a practical, quantitative method for estimating the performance-limiting effects of carbohydrate storage on any runner. It provides a method for endurance runners to assess the degree to which their performances will be limited by their abilities to store glycogen, and enables them to compute personalized, safe, maximum racing paces over endurance distances such as the marathon.

## Methods

### Determining Relative Usage of Fat and Carbohydrate as Fuel Substrates as a Function of Relative Aerobic Intensity (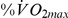)

Aerobic exercise intensity is a relative quantity, frequently measured by the parameter 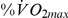: 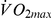 denotes the aerobic capacity of an athlete as measured by the maximum rate at which his or her body can take up oxygen during exercise (typically expressed in milliliters of oxygen, at standard temperature and pressure, per kilogram body mass per minute), and 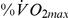 denotes the intensity at which an athlete is working, relative to his or her maximum aerobic capacity, as a percentage of his or her 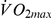. Because aerobic exercise depends on the oxidation of fuel substrates in the working muscles in well defined chemical reactions, the rate at which those muscles take up oxygen from the bloodstream reflects their rate of fuel consumption and hence their maximum power output.

For example, consider a 60-kg elite runner with a 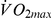 of 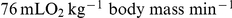, running at an intensity 75% of her 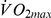. Her rate of oxygen uptake, or 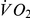, is 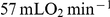 per kilogram body mass, and so her body as a whole is consuming oxygen at a rate of 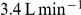 (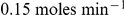).

As indicated in the Results section, the experimental work of Romijn and colleagues [24] provides data on the basis of which the composition of the metabolic mixture consumed during exercise can be estimated as a function of exercise intensity. The functions 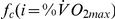 and 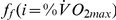, plotted in Figure 1 and described in the Results section, were obtained by fitting quadratic curves to the data presented in that work.

The metabolic fuel mixture being consumed by the athlete in this example is 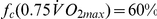 carbohydrate and 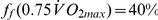 fat. Knowing the proportional contribution of each substrate to the fuel mixture makes it possible to approximate the overall stoichiometry of oxygen use by her muscles. For example, a reaction pathway typical of carbohydrate oxidation involves the complete oxidation of glucose, 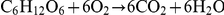, consuming 6 moles of oxygen per mole of carbohydrate, and liberating energy at a density of approximately 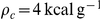 carbohydrate; since glucose has a molar mass of 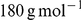, carbohydrate oxidation typically generates approximately 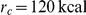 per mole of respired oxygen. On the other hand, a reaction pathway typical of fatty acid oxidation is that of palmitic acid, 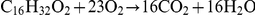, which consumes 23 moles of oxygen per mole of fatty acid, and liberates energy at a density of approximately 
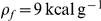
 fatty acid; since palmitic acid has a molar mass of 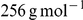, typical fatty acid oxidation generates only approximately 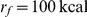 per mole of respired oxygen. These representative computations illustrate the significantly greater efficiency of carbohydrate relative to fat as a fuel for aerobic exercise. Importantly, they also permit the expression of 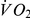 in terms of power output when the composition of the metabolic mixture is known. In general, power output 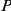 during aerobic exercise (expressed in kilocalories expended per hour, for example) can be inferred from the formula
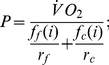
(1)applying this formula in the present example reveals that the runner in question is expending 17 kcal per minute (1020 kcal per hour).

### Running Speed as an Index of Aerobic Intensity (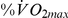)

For a runner whose 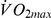 is known, running speed provides a natural way of estimating 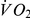, and hence 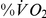: An alternative way of viewing the finding of Margaria and colleagues, cited earlier, is in terms of power expended in running, since a metabolic cost of 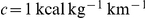, expressed in terms of energy per unit body mass per distance run, is equivalent to 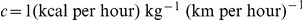, expressed in terms of power per unit body mass per unit running speed (since power is the time rate of change in energy, and speed is the time rate of change in distance). The power expended by a runner is therefore approximately 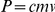, where 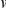 denotes the speed of the runner; equivalently, speed can be expressed in terms of power as 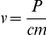. The elite runner of the foregoing example is therefore running at a speed of approximately 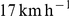 (a pace of 5:53 per mile).

### Estimation of Aerobic Capacity (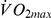)

**Figure 4:**
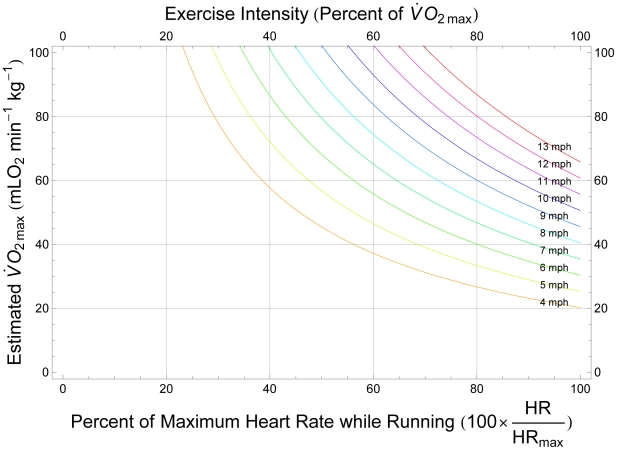
Estimating the aerobic capacity of a typical runner. Computed approximations of 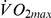 (in terms of milliliters of oxygen per minute per kilogram body mass), as a function of estimated fraction of maximum heart rate while running at a given speed, are shown as a set of colored curves. Each line corresponds to a particular running speed (Orange, 4 mph (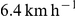); Light Green, 5 mph (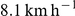); Green, 6 mph (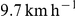); Dark Green, 7 mph (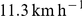); Light Blue, 8 mph (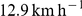); Blue, 9 mph (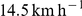); Dark Blue, 10 mph (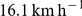); Purple, 11 mph (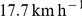); Magenta, 12 mph (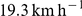); Red, 13 mph (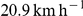)). See the text for a detailed explanation.

A variety of protocols are commonly used to evaluate the 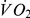 and 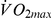 of runners and other athletes. While recreational runners, including the tens of thousands of runners each year who run marathons in cities across the United States and abroad, typically do not have access to the physiologic measurement facilities used to quantify 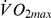 precisely, many do have access to treadmills that permit controlled running at a constant speed. Heart rate, and in particular heart rate as a fraction of maximal heart rate, 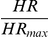, is commonly used as an index of exercise intensity (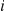 or 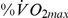) and requires no special apparatus to measure. And while the maximum heart rate, 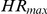, of a particular athlete depends on a variety of factors, it can be estimated using one of many age-dependent formulas, such as the commonly cited one of Fox and Haskell [35] (220 beats per minute minus age in years) or those of Tanaka and colleagues (for active adults, 207 beats per minute minus 0.7 times age in years), which are supported by experiments and by meta-analysis [36]. Since power expended in running is a function of running speed, it is possible to express 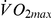 as a function of running speed, 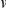, and 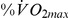 indexed by fractional maximum heart rate, 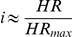, using the approach described in the foregoing analysis:
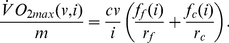
(2)Equation 2 gives 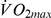 in terms of milliliters of oxygen per minute per kilogram body mass; multiplying by body mass, 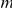, yields total 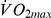, in terms of milliliters of oxygen per minute. Figure 4 illustrates how this formula can be applied to estimate the 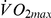 of a runner, and shows a set of computed approximations of 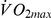 as functions of estimated fraction of maximum heart rate while running at a given speed.

Using Figure 4, the 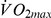 of a runner may be estimated by finding the vertical coordinate, along a colored curve, corresponding to the fraction of his or her maximal heart rate required to sustain the running speed associated with that curve (each colored curve is drawn for a particular running speed, as indicated in the figure legend). For example, consider a thirty-year-old athlete running at 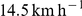 (corresponding to the blue curve in Figure 4). Applying the formula of Tanaka and colleagues, one expects this runner to have a maximum heart rate of approximately 186 beats per minute. Therefore, if he experiences a heart rate of 149 beats per minute, corresponding to 80% of his expected maximum (and therefore to an ‘exercise intensity’ of approximately 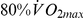), while running at this speed, his estimated 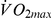 is 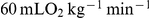, corresponding to the vertical coordinate of the point along the blue curve associated with 80% of maximum heart rate.

### Estimation of the Maximum Aerobic Running Speed

The 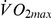 of a runner can be used to estimate his or her maximum aerobically sustainable running speed,
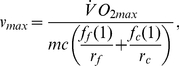
(3)which in turn permits estimation of the relative contributions of carbohydrate and fat to his or her aerobic power output as a function of running speed, since 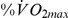 when running at speed 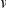 is approximately equal to 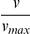. In addition, since the energy required to run a distance 
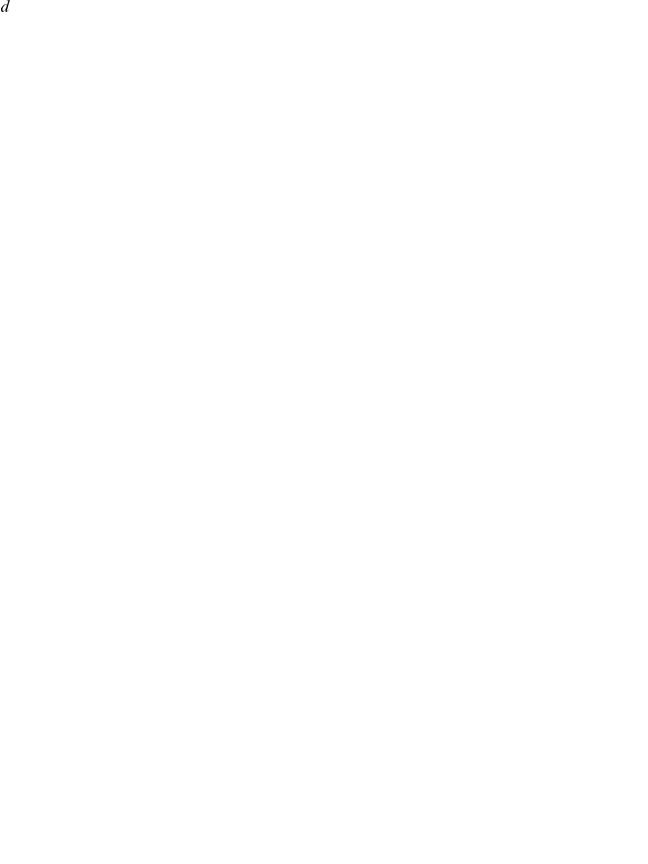
 is 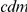 and the fraction of that energy derived from carbohydrate metabolism when running at speed 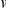 is approximately 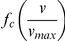,
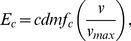
(4)which, for a runner of given mass and 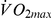 running a fixed distance, depends only on the pace of the runner.

### Estimating Physiologic Glycogen Storage Capacity and the Distance to Run it to Exhaustion

Liver mass is tightly regulated, and normally constitutes approximately 2.5% of total body mass [37]. On the other hand, several studies have examined leg muscle mass in adult populations [38]–[40] and found that it exhibits variability in relation to total body mass, constituting approximately 18.0–22.5% and 14.0–27.5% of total body mass in adult women and men, respectively. Leg muscle mass as a fraction of total body mass therefore accounts for almost all of the variability in the relative size of the maximum carbohydrate storage capacity of an endurance runner, imposing a potential constraint on the maximum speed he or she can sustain over long distances without refueling.

It may be worth noting that Karlsson and Saltin have shown evidence that performance begins to decline before glycogen stores are completely exhausted, even in motivated endurance runners, at leg muscle glycogen concentrations approaching 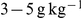 [26]; the effective glycogen reservoir available to a runner may therefore be somewhat smaller than the total glycogen pool. Nevertheless, there is only weak experimental evidence for a time-dependent decline in the rate of carbohydrate metabolism relative to fat metabolism, at constant levels of aerobic intensity. When observed experimentally, the magnitude of such an effect has been on the order of 10%, appearing after 2–3 hours of moderate to strenuous exertion, and has been difficult to distinguish from experimental error [24], [31]. Moreover, careful consideration of physiologic data presented in such studies suggests that such decreases are substantially attributable to declining aerobic output by experimental subjects assigned the physically demanding task of maintaining constant, continuous power output for several consecutive hours. For example, in the study of Bosch and colleagues [31], the correlation coefficients between time-dependent variations in aerobic output and variations in carbohydrate and fat oxidation are 0.59 and 0.79, respectively.

The distance an athlete can run before ‘hitting the wall,’ 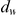, can be expressed as the ratio of energy stored as glycogen per unit body mass to energy consumed as carbohydrate per unit body mass per unit distance, while running at a given intensity. The former is given by the sum of the glycogen densities of the liver and the leg muscles (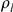 and 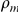, respectively, in terms of kilocalories of glycogen per kilogram tissue), weighted by their relative proportions of total body mass (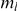 and 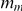, respectively), while the latter is equal to 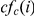. Therefore,
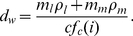
(5)Since most of the variability in 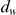 in a given population of runners will be due to characteristics of muscle rather than those of liver, assuming 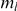 and 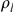 are approximately constant at 2.5% of total body mass and 360 kcal glycogen per kilogram of liver (the maximum physiologic density of liver glycogen), respectively, permits 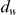 to be considered as a function of leg muscle mass and muscle glycogen density, as well as exercise intensity, as in Figure 3.

The line of reasoning followed here can be extended to estimate the maximal rate of muscle glycogen utilization by a runner, and hence the change in his muscle glycogen density following a long-distance race:
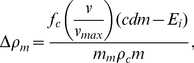
(6)where 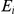 refers to the total carbohydrate energy intake during the race, and 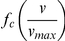 depends on the pace of the runner as well as his aerobic capacity (through the dependence of 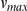 on 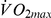). Equation 6 represents a maximal change in density because it assumes that all of the carbohydrate metabolized during exercise is derived from muscle glycogen. In applying this equation to the data of Karlsson and colleagues [26] as described in the Results section, 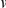 was set to the average race pace of each runner, inferred from his finishing time and the race distance. Karlsson and colleagues permitted each runner to ingest a maximum of 20 g of glucose every 4 km over 30 km but did not monitor the fueling pattern of each runner, so 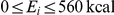 for each of the runners in that study. The parameter 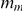 was set to 0.214, a typical value for relative leg muscle mass in men.

### Midrace Fueling Strategies

Exercise-induced hypoglycemia refers to the reduction of plasma glucose levels associated with activities such as endurance running as they deplete total body glycogen stores. The decline of plasma glucose levels causes fatigue, objectively defined as a reduction in muscular force-generating capacity [41]. The onset of muscle fatigue is mediated in part by the central nervous system but is due primarily to metabolic factors, including the depletion of intramuscular fuel reserves [42]. The performance-enhancing capacity of carbohydrate consumption during endurance exercise has been established in a number of studies and review articles [34]. In the context of endurance runners seeking to avoid ‘hitting the wall,’ the modeling approach presented here can be used to compute the amount of carbohydrate a given runner must consume en route in order to ‘push back the wall’ a given distance (beyond, for example, the end of a marathon) and avoid complete glycogen depletion during the race.

As indicated in Equation 4, the carbohydrate energy required by a runner of mass 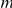 to run distance 
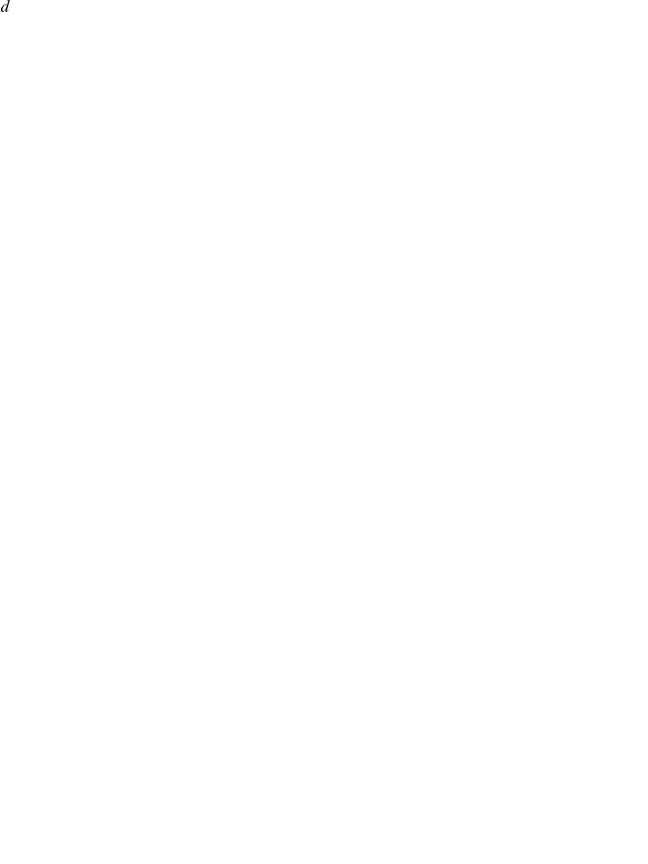
 at relative intensity 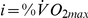 is 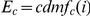. The corresponding mass of carbohydrate is
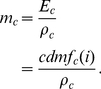
(7)


### Identifying and Quantifying Sources of Error

Several of the techniques and assumptions used to construct and numerically evaluate the model presented here contribute potential sources of error to the derived results. This subsection examines and quantifies the degree to which uncertainty in components of the model contributes to expected errors in its predictions.

First, consider the function 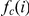, used throughout the model to describe carbohydrate metabolism as a fraction of total energy expenditure, as a function of relative exercise intensity, 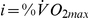. Because 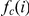 is treated symbolically throughout the derivation of the model equations, the validity of those equations is independent of the degree to which 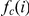 has been accurately characterized. This treatment also facilitates a straightforward sensitivity analysis, in which principal quantities derived from the model are logarithmically differentiated with respect to 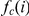 to yield the expected relative (fractional) changes in each of those quantities attributable to relative errors in 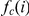.

Consider the expressions for 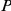, 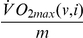, 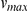, 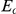, and 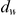 in Equations 1, 2, 3, 4, and 5, respectively. Replacing 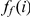 with 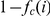 in these equations makes clear that each of these modeled quantities exhibits first-order dependence on 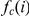 or its inverse. Logarithmic differentiation then reveals that relative errors in 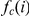 give rise to relative errors of reduced magnitude (positive or negative, respectively) in these quantities. Treating the expression for power output during exercise in Equation 1 in this way yields
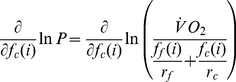
(8)

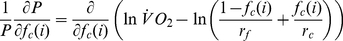
(9)

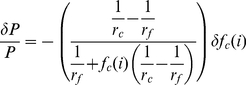
(10)

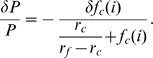
(11)Since 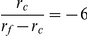 and 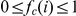,
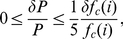
(12)implying that relative errors in 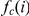 propagate through the model as smaller relative errors in 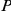. Applying this approach to the expressions for 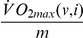 and 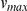 in Equations 2 and 3 yields similar bounds on the relative errors of those quantities:
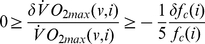
(13)and
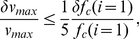
(14)respectively. In stating Inequalities 12, 13, and 14, the magnitudes of variations in the 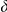-labeled quantities are assumed to be positive; negative variations require reversals of each inequality.

The quantities 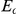 and 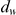 exhibit pure dependence on 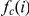 and 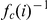, respectively. The expected relative errors in 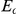 and 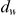 due to their explicit dependence on 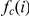 can likewise be derived via logarithmic differentiation, which yields
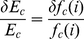
(15)and
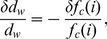
(16)respectively.

The function 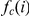 is defined as 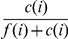, where 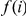 and 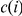 denote the whole-body rates of fat and carbohydrate oxidation, respectively, at a particular intensity. Experimental determination of 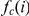 is based on independent measurements of 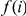 and 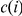 at various intensities, so experimental errors in these two quantities contribute independently to the total error in 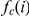. Partial differentiation of 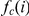 with respect to 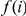 and 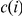 yields the weights assigned to each component in the weighted sum of terms corresponding to the total error in 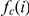:
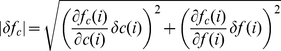
(17)

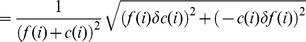
(18)


The particular, fitted functional form of 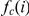 used in this paper in numeric computations is based on data provided in the work of Romijn and colleagues [24]. Values of the standard errors associated with measurements of 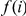 and 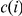 given in that work can be used to derive the total expected error in 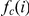 at various aerobic intensities. The corresponding error bars are shown in Figure 1, in which the fitted data for 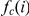 are plotted. The error in 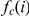 computed according to Equation 18 does not exceed 5.5% at any intensity observed in the study of Romijn and colleagues.

As sensitivity analysis has demonstrated that model errors associated with variations in 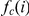 are at most equal in magnitude to, and for some quantities less than or equal to 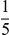 of those variations, the error in any of the modeled quantities attributable to errors in 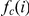 should not exceed 5.5% and should in many cases be closer to 1%.

As a second source of uncertainty, consider the metabolic cost of running, 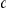. In general the energy cost of running differs from runner to runner, and may also depend on the distance run [43]. Well trained runners typically have greater running economy (lower energy costs of running) than untrained runners; Margaria and colleagues reported the magnitude of this difference to be approximately 5–7% [18]. Remarkably, subsequent studies, such as that of Billat and colleagues [16], have found variations in running economy even among elite runners to be of similar magnitude. Such variations are not consistently correlated with variations in marathon performance, however; Billat and colleagues have demonstrated that the rate of oxygen consumption, 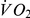 or 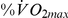, at which the race is run, is a significantly more important factor in determining marathon times.

The individual variability in running economy is of considerably greater magnitude than any error that could be introduced into the model by distinguishing between gross and net energy expenditure, as suggested in the Introduction. An accurate personal model of the gross energy cost of running would have the form 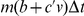, where 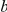 denotes the basal (resting) metabolic rate, approximately 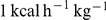, as indicated by Margaria [18]; 
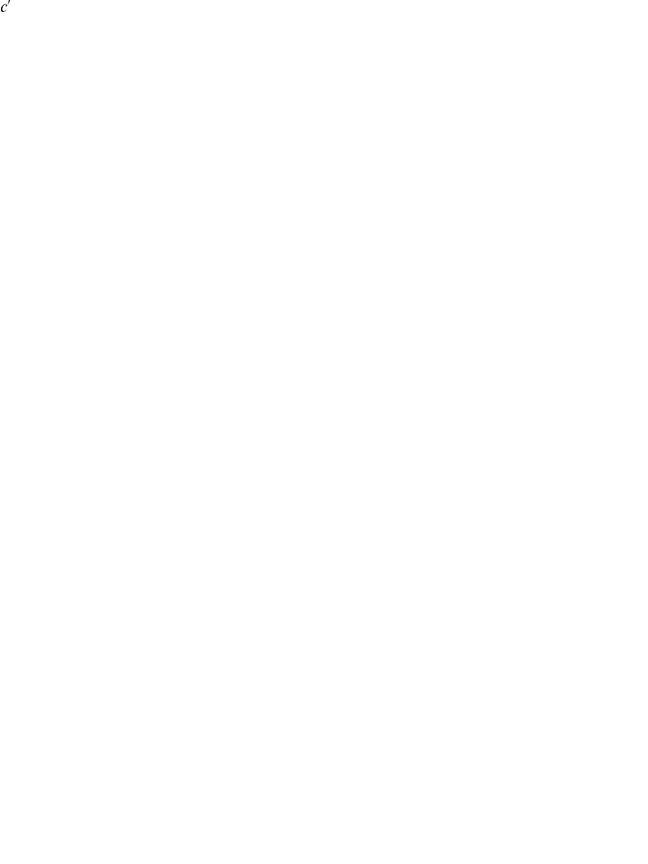
 denotes the net energy cost of running, approximately 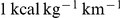; 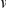 denotes running speed; 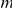 signifies the mass of the runner; and 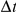 refers to the duration of the run. In this model, the ratio of the basal metabolic contribution to the net energy cost of running is 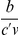, which, numerically, is approximately equal to 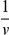 when 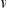 is expressed in kilometers per hour. The modal finishing time in large marathons open to all entrants, as indicated in the Results section, corresponds to a typical running speed 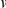 of approximately 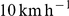. So if basal metabolism consumed all of its energy as carbohydrate, the associated correction to the model presented here would be of order 10%. However, studies such as that of Romijn and colleagues [24] have demonstrated that resting metabolism derives no appreciable energy from muscle glycogen reserves and less than 10% from plasma glucose. Therefore, the error introduced into the model by neglecting 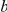 typically has magnitude less than 1%; this error declines further at faster finishing times.

Thirdly, consider the uncertainty associated with methods used to assess aerobic capacity. The accuracy of predictions made using the modeling framework presented here, as applied to a particular runner, depends on the accuracy with which the 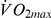 is known for the runner in question. Currently, the most accurate and precise methods of measuring 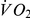 and 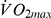 involve quantitative spirometry during progressive exercise protocols [22]. Where the most accurate measurement techniques of contemporary quantitative physiology are available, parameters obtained through such methods can be incorporated into the model equations to generate the most accurate possible results. In the absence of such techniques, to which the typical recreational runner may not have access, principled approximation techniques are extremely useful. To that end, a universally accessible method of approximating 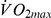 is provided in this paper; errors associated with that method are addressed here.

The method of estimating 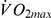 presented in this work belongs to a family of related ‘extrapolation methods’ widely used for many years by athletes, coaches, and trainers. The general approach has been described extensively in the physiology literature (and reviewed by McArdle and colleagues [22], among many others). Specific implementation protocols have been standardized by consensus [28], [44]. The extrapolation methods for measuring 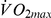 have known shortcomings [22] that may cause them to deviate from spirometric assessments by up to 10–20%. Typical errors may be considerably smaller, however: di Prampero and colleagues [7], for example, employ an extrapolation method similar to the one presented here and find an average ratio of 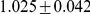 (standard error) on comparing estimates of 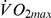 obtained from extrapolation to corresponding measurements obtained via spirometry.

## References

[1] Running USA Road Running Information Center (2010).

[2] Buman MP, Brewer BW, Cornelius AE, Van Raalte JL, Petitpas AJ (2008). Hitting the wall in the marathon: Phenomenological characteristics and associations with expectancy, gender, and running history.. Psychol Sport Exerc.

[3] Buman MP, Brewer BW, Cornelius AE (2009). A discrete-time hazard model of hitting the wall in recreational marathon runners.. Psychol Sport Exerc.

[4] Sjodin B, Svedenhag J (1985). Applied physiology of marathon running.. Sports Med.

[5] Emmett J (2007). The physiology of marathon running: Just what does running a marathon do to your body?. Marathon and Beyond.

[6] Locksley R (1980). Fuel utilization in marathons: Implications for performance (Medical staff conference, University of California, San Francisco).. West J Med.

[7] di Prampero PE, Atchou G, Brückner JC, Moia C (1986). The energetics of endurance running.. Eur J Appl Physiol Occup Physiol.

[8] Ward-Smith AJ (1985). A mathematical theory of running, based on the first law of thermodynamics, and its application to the performance of world-class athletes.. J Biomech.

[9] Péronnet F, Thibault G (1989). Mathematical analysis of running performance and world running records.. J Appl Physiol.

[10] Joyner MJ (1991). Modeling: optimal marathon performance on the basis of physiological factors.. J Appl Physiol.

[11] Billat V, Bocquet V (1999). Bioenergetics approach of female marathon best performance.. Sci Sports.

[12] Bocquet V, Billat V (1999). Mathematical and physiological models of human performance.. Sci Sports.

[13] di Prampero PE (2003). Factors limiting maximal performance in humans.. Eur J Appl Physiol.

[14] McGregor SJ, Weese RK, Ratz IK (2009). Performance modeling in an olympic 1500-m finalist: A practical approach.. J Strength Cond Res.

[15] Jones AM (2006). The physiology of the world record holder for the women's marathon.. Int J Sports Sci Coach.

[16] Billat VL, Demarle A, Slawinski A, Paiva M, Koralsztein JP (2001). Physical and training characteristics of top-class marathon runners.. Med Sci Sports Exerc.

[17] Arrese LA, Izquierdo DM, Serveto Galindo JR (2005). Physiological measures associated with marathon running performance in high-level male and female homogeneous groups.. Int J Sports Med.

[18] Margaria R, Cerretelli P, Aghemo P, Sassi G (1963). Energy cost of running.. J Appl Physiol.

[19] Mayhew JL (1977). Oxygen cost and energy expenditure of running in trained runners.. Br J Sports Med.

[20] Acheson KJ, Schutz Y, Bessard T, Anantharaman K, Flail JP (l988). Glycogen storage capacity and de novo lipogenesis during massive carbohydrate overfeeding in man.. Am J Clin Nutr.

[21] Hultman E, Nilsson LH (1973). Liver glycogen content in man in the postabsorptive state.. Scand J Clin Lab Invest.

[22] McArdle WD, Katch FI, Katch VL (2010). Exercise Physiology: Nutrition, Energy, and Human Performance.

[23] Fairchild TJ, Fletcher S, Steele P, Goodman C, Dawson B (2002). Rapid carbohydrate loading after a short bout of near maximal-intensity exercise.. Med Sci Sports Exerc.

[24] Romijn AJ, Coyle EF, Sidossis LS, Gastaldelli A, Horowitz JF (1993). Regulation of endogenous fat and carbohydrate metabolism in relation to exercise intensity and duration.. Am J Physiol.

[25] Berg JM, Tymoczko JL, Stryer L (2002). Biochemistry.

[26] Karlsson J, Saltin B (1971). Diet, muscle glycogen, and endurance performance.. J Appl Physiol.

[27] Sabhapandit S, Majumdar SN, Redner S (2008). Crowding at the front of marathon packs.. J Stat Mech arXiv.

[28] American College of Sports Medicine (2000). Guidelines for Exercise Testing and Prescription.

[29] Pollock ML (1973). The quantification of endurance training programs.. Exerc Sport Sci Rev.

[30] Arkinstall MJ, Bruce CR, Clark SA, Rickards CA, Burke LM (2004). Regulation of fuel metabolism by preexercise muscle glycogen content and exercise intensity.. J Appl Physiol.

[31] Bosch AN, Dennis SC, Noakes TD (1993). Influence of carbohydrate loading on fuel substrate turnover and oxidation during prolonged exercise.. J Appl Physiol.

[32] Tsintzas OK, Williams C, Boobis L, Greenhaff P (1996). Carbohydrate ingestion and single muscle fiber glycogen metabolism during prolonged running in men.. J Appl Physiol.

[33] Thomas JE (1957). Mechanics and regulation of gastric emptying.. Physiol Rev.

[34] Tsintzas K, Williams C (1998). Human muscle glycogen metabolism during exercise: Effect of carbohydrate supplementation.. Sports Med.

[35] Fox SM, Haskell WL, Naughton JP (1971). Physical activity and the prevention of coronary heart disease.. Ann Clin Res.

[36] Tanaka H, Monahan KD, Seals DR (2001). Age-predicted maximal heart rate revisited.. J Am Coll Cardiol.

[37] Valentin J (2003). Basic Anatomical and Physiological Data for Use in Radiological Protection: Reference Values, volume 32 of Annals of the International Commission on Radiological Protection.

[38] Heymsfield SB, Smith R, Aulet M, Bensen B, Lichtman S (1990). Appendicular skeletal muscle mass: measurement by dual-photon absorptiometry.. Am J Clin Nutr.

[39] Wang W, Wang Z, Faith MS, Kotler D, Shih R (1999). Regional skeletal muscle measurement: evaluation of new dual-energy x-ray absorptiometry model.. J Appl Physiol.

[40] Levine JA, Abboud L, Barry M, Reed JE, Sheedy PF (2000). Measuring leg muscle and fat mass in humans: comparison of CT and dual-energy x-ray absorptiometry.. J Appl Physiol.

[41] Nybo L (2003). CNS fatigue and prolonged exercise: Effect of glucose supplementation.. Med Sci Sports Exerc.

[42] Kent-Braun JA (1999). Central and peripheral contributions to muscle fatigue in humans during sustained maximal effort.. Eur J Appl Physiol Occup Physiol.

[43] Brueckner JC, Atchou G, Capelli C, Duvallet A, Barrault D (1991). The energy cost of running increases with the distance covered.. European J Appl Physiol.

[44] Golding LA (2000). YMCA Fitness Testing and Assessment Manual.

